# Research Progress Towards Poliovirus Virus-like Particle Vaccines: A Review

**DOI:** 10.3390/vaccines14030216

**Published:** 2026-02-27

**Authors:** Taoli Han, Jinbo Xiao, Shiyao Zhang, Tongyue Su, Yinuo Liu, Yong Zhang

**Affiliations:** 1 National Key Laboratory of Intelligent Tracking and Forecasting for Infectious Diseases (NITFID), National Institute for Viral Disease Control and Prevention, Chinese Center for Disease Control and Prevention, Beijing 102206, China; hantaoli0702@163.com (T.H.);; 2 National Laboratory for Poliomyelitis, WHO WPRO Regional Polio Reference Laboratory, National Institute for Viral Disease Control and Prevention, Chinese Center for Disease Control and Prevention, Beijing 102206, China; 3 National Health Commission Key Laboratory of Microbial Genomics, National Health Commission Key Laboratory for Biosafety, National Institute for Viral Disease Control and Prevention, Chinese Center for Disease Control and Prevention, Beijing 102206, China; 4 Beijing Chaoyang Center for Disease Control and Prevention, Beijing 100021, China; 5 School of Public Health, Hebei University, Baoding 071000, China

**Keywords:** poliovirus, virus-like particles (VLPs), vaccine, polio eradication

## Abstract

Poliovirus (PV), a historically significant enterovirus responsible for severe paralytic diseases, has seen its incidence dramatically reduced through widespread vaccination efforts, propelling global eradication initiatives. Despite the success of traditional oral poliovirus vaccines (OPVs) and inactivated poliovirus vaccines (IPVs), challenges such as vaccine-derived virus reversion and biosafety concerns during vaccine production persist. Virus-like particle (VLP) vaccines, which mimic native viral structures without containing viral genomes, offer enhanced safety profiles and robust immunogenicity, positioning them as promising candidates for next-generation poliovirus vaccines, especially in the post-certification era. This review systematically summarizes current progress in poliovirus VLP vaccine research, including the diverse expression systems employed for VLP production, strategies for peptide assembly and stabilization, and evaluations of antigenicity and immunogenicity. Additionally, it highlights structural analyses utilizing cutting-edge cryo-electron microscopy. By integrating recent developments in genetic engineering, structural biology, and immunology, this article discusses the advantages and challenges associated with poliovirus VLP vaccines and explores future directions aimed at supporting the global goal of a poliovirus-free world. This comprehensive overview aims to provide a theoretical foundation and technical guidance to facilitate the development and deployment of safer and more effective poliovirus vaccines.

## 1. Introduction

Poliovirus (PV) is the etiological agent of poliomyelitis. It can cause irreversible paralysis and even death, primarily affecting children under 5 years of age. The Global Polio Eradication Initiative (GPEI), launched in 1988, has substantially reduced global poliovirus incidence: wild-type poliovirus Type 2 (WPV2) and wild-type poliovirus Type 3 (WPV3) were declared successfully eradicated in 2015 and 2019, respectively [1]. However, wild-type poliovirus Type 1 (WPV1) remains endemic in Afghanistan, Pakistan, and other regions, while the emergence of vaccine-derived polioviruses (VDPVs) has posed new challenges to eradication efforts [2]. By 2025, all WHO regions except the Eastern Mediterranean Region (EMR) have interrupted indigenous WPV1 transmission and been certified for elimination, with the EMR retaining endemic WPV1 in Afghanistan and Pakistan [2,3]. As of 22 October 2025, a total of 38 global cases of wild WPV1 have been reported, with 9 in Afghanistan and 29 in Pakistan. This represents a notable decline compared with the 62 cases documented during the same period in 2024 [3]. Moreover, 151 cVDPV2 cases (13 countries) have been reported globally, compared with 182 cases (16 countries) in the same period in 2024. Transmission persists in low-immunization areas including northern Nigeria, the Lake Chad Basin, the Horn of Africa (south-central Somalia, Ethiopia), and Yemen. Three global VDPV1 cases have been reported (one each in Algeria, the DRC and the Lao People’s Democratic Republic), with environmental detections in Djibouti. VDPV3 was detected in Guinea between 2024 and 2025, with no new cases since March 2025. Limited cases were also reported in Cameroon and Chad in 2025, and targeted outbreak response measures are ongoing to curb any further spread [3].

PV is a positive-sense single-stranded RNA virus. Based on the 2024 release of the ICTV (International Committee on Taxonomy of Viruses) taxonomy, PV is classified as *Enterovirus coxsackiepol* (ICTV Taxon ID: 202401985), which belongs to the family *Picornaviridae*, order *Picornavirales* (https://ictv.global/taxonomy, accessed on 18 February 2026). It encompasses three distinct serotypes, namely Type 1, Type 2, and Type 3. The induced protective immunity is serotype-specific and lacks cross-protective efficacy across serotypes. The open reading frame (ORF) of PV is translated into a single polyprotein, which is proteolytically cleaved into mature viral proteins. Notably, protease 3C (cleaving the P2/P3 regions) and its precursor 3CD (cleaving the structural precursor P1 and participating in the initiation of viral RNA replication by polymerase 3D) play key roles [4,5,6]. Processing of P1 generates the capsid proteins VP0, VP3, and VP1, and VP0 is further cleaved into VP2 and VP4 (possibly driven by viral genome encapsidation), which ultimately assemble into mature 30-nm icosahedral viral particles containing 60 copies each of VP1 through VP4. During viral infection, empty capsids (ECs) lacking the viral genome are also produced. These ECs, which resemble mature viral particles but contain uncleaved VP0, can serve as virus-like particle (VLP) vaccines to replace existing PV vaccines. However, wild-type ECs are unstable and are prone to conformational expansion and conversion from the native D-antigen to the non-native C-antigen. The latter has a weak ability to induce neutralizing antibodies and cannot provide long-term protective immune responses [7,8,9].

At present, two PV vaccine types—oral poliovirus vaccines (OPVs) and inactivated poliovirus vaccines (IPVs)—are widely used against all three serotypes [10]. OPV is low-cost, easy to administer, and induces gastrointestinal mucosal immunity to prevent PV transmission. It may cause vaccine-associated paralytic poliomyelitis (VAPP) in humans at low incidence rate and may also regain neurovirulence via reversion mutations to form circulating vaccine-derived PV (cVDPV) [10,11]. A further strategy to overcome the drawbacks of OPV is also addressed herein, with the mention of novel oral poliovirus Type 2 (nOPV2)—which boasts genetic stability and improved production safety—but it still cannot serve as a definitive solution. IPV avoids the risks of neurovirulence reversion but cannot block fecal–oral transmission, as it does not induce gastrointestinal mucosal immunity [12]. Both vaccines rely on the large-scale culture and handling of live infectious PV, creating accidental release risks or necessitating cold-chain storage and transportation [13]. This underscores the imperative for next-generation live-virus-free PV vaccines.

**Figure 1:**
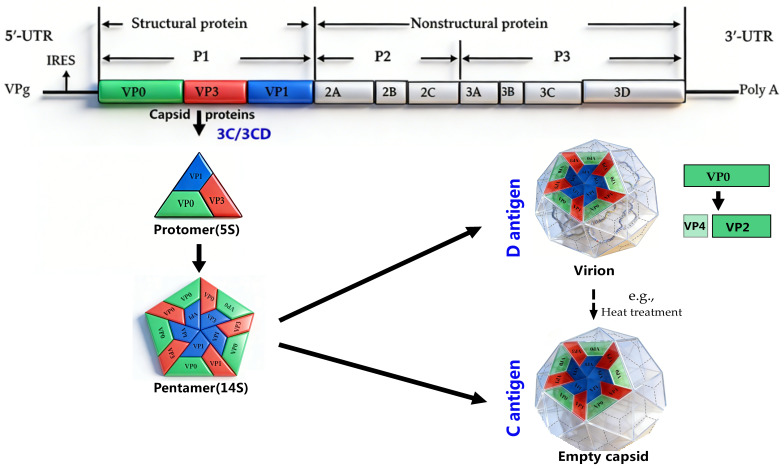
Schematic diagram of poliovirus’s genomic structure and VLP assembly [8,16,17]. This figure was created by the authors based on [8,16,17]. The PV genome is a single positive-sense RNA molecule which includes a 5′ UTR containing an internal ribosomal entry site (IRES), a 3′ UTR, and a poly(A) tail. The open reading frame (ORF) encodes a polyprotein processed into structural (P1) and nonstructural (P2, P3) regions. P1 is cleaved into VP0, VP3, and VP1 by the 3CD protease. A 5S protomer is assembled from one copy of VP0, VP3, and VP1, and five protomers are assembled into a 14S pentamer. In the absence of viral genomic RNA, 80S empty particles (C-antigen) are self-assembled from these pentamers. After viral genome encapsidation, a 150S particle is formed; VP0 is further cleaved into VP4 and VP2, and mature 160S virions (D-antigen) are consequently formed. Heating or improper handling of viral particles can induce the conversion from D-antigens to C-antigens.

Accordingly, researchers are focused on developing virus-like particles (VLPs) as a more promising vaccine platform. VLPs mimic the structure of an authentic infectious virus but lack any viral genetic material, and have been validated as a safe, effective vaccine platform for some viruses (e.g., human papillomavirus and human hepatitis B virus) [14,15]. This characteristic significantly provides a safer alternative to traditional vaccines and addresses the reversion risk in vaccinated populations [16] (Figure 1).

Here, we will summarize current progress in the development of PV VLP vaccines, detailing the expression systems, structural stabilization strategies, and immunological characteristics of these novel vaccine candidates. By synthesizing recent findings and advances, this study aims to provide a comprehensive overview of PV VLP-based vaccine development and its implications for global polio eradication efforts. Beyond PV, VLP technology holds promise for developing vaccines against other viral pathogens, emphasizing the versatility and potential of this innovative vaccine platform in addressing global health challenges [17].

## 2. Expression System of Poliovirus VLPs

### 2.1. Baculovirus–Insect Cell Expression System

The baculovirus–insect cell expression system is currently the most mature and high-yielding platform for PV VLP production, with advantages including robust protein expression capacity, support for multi-gene co-expression, and easy process scale-up, making it suitable for large-scale manufacturing [18]. Studies have successfully produced PV VLPs of all three serotypes (Type 1, Type 2, and Type 3) using this system [16]. Although PV capsid proteins are not glycosylated per se, the high protein expression capacity and proper folding environment inherent to the baculovirus–insect cell system are crucial for generating conformationally correct D-antigenic VLPs [19]. VLPs produced by this system have been extensively evaluated in terms of antigenicity, thermostability, and immunogenicity. For instance, stabilized PV VLPs of the three serotypes derived from different recombinant expression systems (including the insect cell system) were subjected to immunization experiments in female Wistar rats. The results showed that on Day 28 post-immunization, the neutralizing antibody titers in the serum reached 1:3200, 1:2800, and 1:3000, which were significantly higher than that of 1:2500 in the control IPV group (*p* < 0.05). In addition, the antibodies’ persistence in animals from the VLP groups exceeded 12 weeks, demonstrating comparable or even superior immunogenicity to that of the current IPV [16].

### 2.2. Yeast Expression System (Pichia pastoris)

The yeast expression system, particularly *Pichia pastoris* (with e.g., high expression, secretability, eukaryotic folding, and modification), is a highly advantageous platform for PV VLP production, owing to its high expression efficiency, scalability, and capacity for eukaryotic post-translational modifications [20,21]. Similar to other expression systems, the yeast expression system can co-express the structural precursor protein P1 and the viral protease 3CD, a key characteristic for efficient VLP assembly, mimicking natural viral capsid formation [16]. The existing research shown that *Pichia pastoris* is a viable expression system for producing D-antigenic PV VLPs, which not only exhibit multiple characteristics similar to those of the current vaccine but also have the potential to outperform it [8]. For example, the ratio of D-antigen to C-antigen of PV-3 SC8 VLPs expressed in the *Pichia pastoris* system is approximately 2.5:1, whereas that of wt PV-3 VLPs is around 1:1. Moreover, PV-3 SC8 VLPs retained 50% of D-antigens at a higher temperature than IPV (53 °C vs. 51 °C); notably, 48% of D-antigens remained detectable in PV-3 SC8 VLPs even at 55 °C, whereas D-antigen was undetectable in IPVs at this temperature.

### 2.3. Mammalian Cell Expression System

Mammalian cell expression systems (e.g., BHK-21 cells) provide a protein folding and modification environment that most closely mimics that of native viruses, and thus the VLPs produced may possess superior antigenic conformation [22]. For instance, across all tested doses except the highest level (proportion of a single human dose: 100%), a higher proportion of animals seroconverted following immunization with stabilized PV3 SC8 VLPs expressed in BHK-21 cells than with IPV in immunized (i.m.) rats. Furthermore, PV3 SC8 VLPs exhibited markedly enhanced D-antigen thermostability, retaining 50% D-antigenicity at temperatures above 60 °C [13]. Additionally, the characterized capsid mutant of PV-1 Mahoney (PV57δ) has been shown to enhance D-antigens’ stability in mammalian cell cultures [23,24].

Evidence suggests that the thermostabilized PV3 variant of VLPs expressed in HEK293 cells were verified to exhibit a native D-antigen conformation. Experimental data demonstrated that the neutralizing antibody titer induced by this variant reached 1:6400, which was significantly higher than that of the control group (1:3200), and showed no significant difference from that of the current IPV, indicating equivalent immunogenic efficacy Cryo-electron microscopy (cryo-EM) analysis revealed that these VLPs were indistinguishable from the empty capsids generated during natural infection and contained similar stabilizing lipid pocket factors within the VP1 β-barrel structure [13]. Such factors are present in mammalian cell-expressed VLPs but may not be acquired in alternative expression systems, which highlights the potential of mammalian cell systems to recapitulate the key features of native viral particles.

It is unlikely to be the preferred system for post-eradication PV vaccines (e.g., due to MVA vector-based expression), but mammalian-derived VLPs serve as a gold standard for comparison with VLPs from other recombinant systems and support alternative vaccine strategies. Despite the higher costs, their capacity to produce structurally authentic and immunologically potent VLPs makes them highly advantageous for developing next-generation PV vaccines.

### 2.4. Other Expression Systems

In addition to the expression systems above, plant-based platforms also have been explored for PV VLP production, with unique advantages for scalability and biosafety. Similarly, plant expression systems (e.g., *Nicotiana benthamiana* [25]) have been investigated as VLP biofactories, leveraging rapid growth, the low risk of human pathogen contamination, and large-scale cultivation potential. Both inherently reduce the biosafety concerns associated with handling live PV, as VLPs lack viral genomes and are non-replicative/non-infectious. Plant-based vaccines can be generated via stable plastid/nuclear genome transgene delivery, transient nuclear transformation, or RNA viral expression systems [26].

In conclusion, these application of PV VLP vaccines remains largely at the laboratory stage. The recombinant trivalent poliovirus vaccine (Sf-RVN cell-derived, trivalent VLP-Polio) developed by CanSino Biologics has launched Phase I/II clinical trials in Indonesia, with the aim of evaluating its safety profile and immunogenicity in a cohort of infants and young children within designated age groups after vaccination (https://www.cansinotech.com.cn/detail-4558, accessed on 28 December 2025). Key challenges include optimizing the expression vectors, enhancing protein yield, and ensuring consistent particle assembly/stability. For instance, achieving adequate P1 (structural precursor protein) and 3CD (viral protease) expression in these heterologous hosts requires fine-tuning the transcriptional and translational control elements to balance synthesis and mitigate cytotoxicity. VLPs produced by this system have been extensively evaluated in terms of antigenicity, thermostability, and immunogenicity, demonstrating comparable or even superior immunogenicity to that of current IPV [16].

## 3. Some 3CD Toxicity Reduction Strategies

It should be noted that 3CD exhibits cytotoxicity, which impairs host cell viability and reduces VLP yield, a prevalent phenomenon [23,27]. To tackle this challenge, various genetic regulatory strategies have been developed to reduce 3CD-induced cytotoxicity in expression systems.

These include using separate transcriptional units for P1 and 3CD, as well as molecular elements to enable controlled polycistronic expression. For instance, the *Thosea asigna virus 2A (TaV 2A)* peptide was employed to mediate co-translational “cleavage” (ribosome skipping) from a single mRNA, yielding two proteins, with the upstream one containing a C-terminal tag [28,29]. Notably, protease-independent production strategies have also been developed, in which the P1 sequence is engineered to produce porcine teschovirus 2A (P2A) peptides that terminate translation between capsid proteins, obviating 3CD co-expression and eliminating the associated cytotoxicity. These protease-independent VLPs retain their native antigenicity and structural integrity, and exhibit thermostability exceeding physiological temperatures—an advantage for vaccine storage and distribution [27].

Alternatively, internal ribosome entry site (IRES) from Rhopalosiphum padivirus (RhPV)—structured RNA elements that permit cap-dependent translation initiation—was used to drive separate translation of two native, untagged proteins from a bicistronic mRNA [30,31]. Each strategy fine-tunes 3CD expression to levels supporting proper capsid protein processing without overburdening the host cell machinery (wt PV1: wild-type poliovirus Type 1, PV57δ: mutant of PV-1 Mahoney) [23]. For 3CD expression regulation, previous studies inserted a TaV 2A peptide (between the P1 terminus and the 3CD start site) or an RhPV IRES (between P1 and 3CD). TaV 2A, a highly efficient cleaving peptide, maintains P1:3CD at 1:1–2:1 via differential translation reinitiation [32]. RhPV IRES affects 3CD levels more significantly (IRES-mediated translation is 5–20-fold less efficient than cap-dependent translation [32]). Moreover, both TaV 2A and RhPV IRES mediate 3CD’s translation in *Pichia pastoris*.

**Figure 2:**
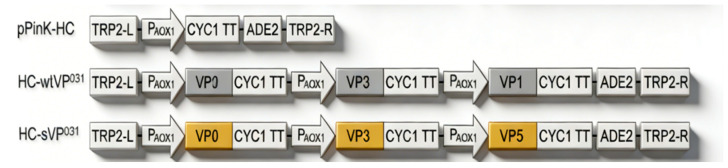
PV2 wtVLP and sVLP co-expression strategy [33]. This figure is reproduced from [33]. TRP2-L and TRP2-R, the up- and downstream parts of the TRP region; PAOX1, AOX1 promoter; CYC1 TT, CYC1 transcription termination region; ADE2, expression cassette encoding phosphoribosylaminoimidazole carboxylase, used as the selection marker.

Furthermore, one study has also indicated that PV2 wtVLP and sVLP can be efficiently produced in *Pichia pastoris* yeast through a novel strategy—directly co-expressing the VP0, VP1, and VP3 capsid subunits [33]. On the one hand, the P1/3CD co-expression approach relies on the 3CD-mediated cleavage of P1 to produce the VP0,VP1, and VP3 subunit proteins, but this process is somewhat inefficient or incomplete; the VP0/VP1/VP3 co-expression strategy omits this step [13,33] (Figure 2), boosting VLP assembly efficiency while circumventing 3CD toxicity to enhance yeast growth and target protein accumulation (e.g., a yield of 0.2 mg purified VLP per gram of yeast, wet weight, and 7.33 DU per µg of protective D-antigen protein). Importantly, purified sVLPs exhibited protective D-antigenicity (7.33 DU/µg protein), corresponding to 1466 DU per gram of sVLP-expressing yeast, equivalent to 183 human doses of commercial PV2 IPV (8 DU per dose) [33].

## 4. Stabilization Strategies for Poliovirus VLPs

PV-VLPs, as metastable protein assemblies, suffer from physicochemical instability, which impairs vaccine efficacy and shelf life. Their native D-antigen conformation is required for inducing neutralizing antibodies [33], but a lack of genomic RNA scaffolding causes a transition from 160S D to 135S A particles with epitope loss [34,35]. Weak pentameric/intercapsid interactions and high thermosensitivity (irreversible disruption > 37 °C) further induce disassembly and restrict cold-chain-independent delivery [35,36]. Elucidating and resolving these issues is key to developing stable, efficacious PV-VLP vaccines.

### 4.1. Targeted Mutagenesis of Key Amino Acid Residues

Site-directed mutagenesis represents a classical yet highly effective rational design strategy for enhancing protein stability. Its core principle lies in the precise identification of key amino acid residues that sustain pentameric interfaces and icosahedral symmetric contacts, via the analysis of high-resolution crystal structures or cryo-electron microscopy (cryo-EM) structures of virus-like particles [37,38]. Typically localized at subunit interfaces, these residues stabilize the overall assembly of particles through hydrophobic interactions, hydrogen bonds, or salt bridges. Table 1 summarizes partial identified mutations with the potential to enhance capsids’ stability, along with their origins and topological locations [39].

### 4.2. Introducing Intramolecular or Intermolecular Disulfide Bonds

The introduction of engineered disulfide bonds serves as a robust covalent crosslinking strategy that markedly enhances the mechanical rigidity and stability of protein complexes [40]. For VLPs, a pair of cysteine (Cys) residues can be rationally designed and incorporated, either between adjacent protein subunits or across distinct structural domains within a single subunit, on the basis of in silico modeling and structural analysis [41,42]. Under oxidative conditions, the thiol groups (-SH) of these two cysteines form covalent disulfide bonds (-S-S-), thereby locking the originally non-covalently associated components in place [43,44]. However, this strategy demands meticulous design to avoid disrupting the native protein conformation or masking critical antigenic epitopes [45]. This underlying principle has been validated in synthetic polymer materials and provides valuable insights into the rational engineering of VLPs. Thus, the rational design and introduction of disulfide bonds represent a highly promising approach for developing ultra-stable VLP-based vaccine candidates.

### 4.3. Adjuvants and Small-Molecule Stabilizers

Recent research has confirmed that specific adjuvants and small-molecule stabilizers improve VLPs’ stability and efficacy. Notably, glutathione (GSH) binds to VLPs’ interprotomer pockets to stabilize the capsids’ native conformation via key protein–protein interactions, avoiding immunogenicity loss from premature disassembly or conformational changes. Yeast-derived thermostabilized PV2 VLPs (sVLPs) show higher D-antigen levels and thermostability than wild-type VLPs, inducing stronger neutralizing antibody responses with adjuvants (e.g., aluminum hydroxide) [33].

In addition to natural molecules like GSH, sulfonamide-related synthetic small molecules have been explored to further stabilize VLPs by over-stabilizing the interprotomer pockets. For example, a capsid-binding antiviral pleconaril inhibits rhinovirus and PV infections by binding to the viral capsid and blocking RNA release, illustrating the therapeutic potential of small molecules that stabilize viral structural proteins [46]. Integrating these adjuvants and small-molecule stabilizers into VLP vaccine formulations offers significant practical advantages. Furthermore, efficient yeast-based production of stabilized VLPs, combined with these agents, supports scalable manufacturing meeting global vaccine demand, while minimizing the biosafety risks associated with live virus handling [47]. Overall, future research focusing on optimizing these stabilizers and elucidating their precise molecular interactions with VLPs will be crucial for advancing PV vaccine formulations and could serve as a model for other picornavirus VLP vaccines [17].

### 4.4. Protease-Dependent and Protease-Independent Production Technologies

Traditional poliovirus VLP production depends on 3CD protease-mediated cleavage of the P1 precursor protein, mimicking native assembly (3CD cleaves P1 into VP1–VP4 for icosahedral capsid self-assembly), but 3CD-induced cytotoxicity (non-specific host protein cleavage and cellular stress/apoptosis, especially in eukaryotes) impairs cell viability and VLP yield, so alternative strategies are needed to enhance the safety and efficiency of this foundational method [27].

In response to the challenges of protease-dependent poliovirus VLP production, protease-independent expression strategies (e.g., using P2A self-cleaving peptides to mediate co-translational separation of capsid proteins via ribosomal peptide bond formation skipping at P2A sites in a single open reading frame, thus generating discrete capsid proteins without proteolytic cleavage) have been developed to reduce cytotoxicity, enhance host cells’ viability and improve VLP yield, and stabilized PV-1 VLPs with native antigenicity and proper icosahedral morphology have been successfully generated via P2A-mediated cleavage in the *Pichia pastoris* expression system, while only VP3 maintained antigenic integrity and tolerated the P2A tag, highlighting the important role of protein order and sequence context in construct design [27].

Additionally, a study has also demonstrated that PV2 wild-type VLPs (wtVLPs) and stabilized VLPs (sVLPs) can be efficiently manufactured in *Pichia pastoris* yeast via an innovative strategy: the direct co-expression of the VP0, VP1, and VP3 capsid subunits [33]. In summary, continued refinement and application of these protease-independent technologies will be essential for advancing poliovirus VLP vaccines and potentially other enterovirus vaccines [27,43].

## 5. Antigenicity and Immunogenicity of Poliovirus VLPs

### 5.1. Antigen Conformation and Its Immunological Significance

The antigenic profile of PV is defined by two distinct conformational states that emerge during maturation, each with profound implications for immunogenicity. The D-antigen (native 160S infectious virions) present at maturity, is the principal target for protective immunity. It presents conformation-dependent neutralizing epitopes (e.g., the VP1 canyon G-H loop, the VP2 EF loop–VP1 B-C loop complex) whose structural integrity is maintained by factors like lipids within the VP1 pocket [48]. In contrast, the C-antigen (empty 80S/135S capsids or thermally denatured particles) is associated with conformationally altered or expanded particles, including empty capsids or heat-denatured virions. This state exhibits markedly weaker immunogenicity, as the structural expansion dislocates the critical neutralizing epitopes, exposing only non-neutralizing linear sequences (e.g., VP1 the N-terminus) [13].

**Figure 3:**
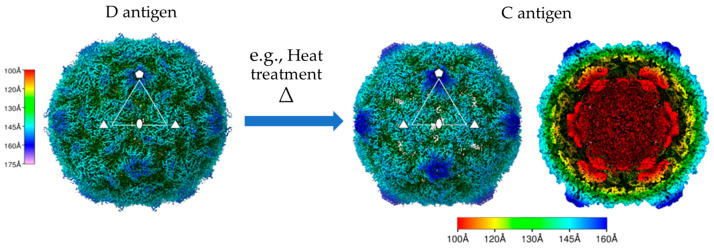
Cryo-EM structures of D-antigen and C-antigen particles of poliovirus Type 1 (Mahoney strain) [9]. This figure is reproduced from [9]. D-antigen: Radially colored isosurface of refined particles at high contour (160S (**left panel**)); C-antigen: Radially colored isosurface of early 135S particles at high contour (σ = 5), showing an opening at the 2-fold axis (**right panel**). Scale bar indicates the radial range corresponding to each color.

The antigenic fidelity of VLPs, crucial for their efficacy as vaccine candidates, fundamentally depends on preserving the native D-antigenic conformation. Structural studies, notably by cryo-electron microscopy (cryo-EM), confirm that stabilized D-state VLPs faithfully present the native epitope landscape [8,9] (Figure 1 and Figure 3). Conversely, conversion to the C state—triggered by mechanisms such as VP0 cleavage loss, pocket factor dissociation, or axis channel opening—results in the loss of these key neutralizing epitopes, thereby compromising the induction of protective antibodies [49].

This dynamic D-to-C conversion is therefore a critical regulator of viral biology. It governs not only particles’ maturation and stability but also represents a potential mechanism for immune evasion, ultimately shaping the complex interplay between viral pathogenicity and the host’s immune responses. In addition, non-competitive sandwich ELISA can be used to measure the content of D- and C-antigens [23].

Conformationally stabilized VLPs that preserve the native D-antigenic state elicit robust neutralizing antibody responses, demonstrating superior immunogenicity compared with C-state or certain conventional inactivated vaccines [13,33]. For instance, thermostabilized PV3 VLPs produced in mammalian cell systems maintained the native D-state and induced neutralizing antibody titers comparable with those of current IPVs, underscoring the immunological advantage of stable D-state VLPs [13]. Similarly, yeast-expressed stabilized PV2 VLPs exhibited enhanced thermostability and higher D-antigen content relative to wt VLPs, which correlated with improved neutralization titers in murine models [33].

The structural integrity of the D-antigen is maintained through specific capsid protein mutations that prevent a conformational shift to the expanded, immunologically inferior C form. Cryo-EM analyses confirm that stabilized VLPs closely resemble native virions, including the retention of key stabilizing factors (e.g., lipidic pocket factors within the VP1 β-barrel) that are essential for antigenic fidelity [8,13].

Thermally stable VLPs offer particular advantages for vaccine deployment in resource-limited settings, as they retain immunogenicity after exposure to elevated temperatures, outperforming less stable VLPs and some conventional vaccine formulations [33]. This antigenic stability is critical not only for inducing potent neutralizing antibodies but also for ensuring consistent vaccine efficacy and safety in the post-eradication era, where live-virus-based vaccines pose biosafety risks.

Advances in recombinant expression systems (e.g., *Pichia pastoris*, mammalian cells) have enabled the production of VLPs that preserve the native D-antigen conformation without the need for live virus culture, providing safer and more scalable vaccine candidates [8,47]. Furthermore, innovative production strategies (e.g., protease-independent methods) allow the generation of native-like antigenic particles while circumventing cytotoxicity issues, thereby enhancing the feasibility of VLP-based vaccines [27].

Collectively, these findings highlight the central importance of maintaining the D-antigenic conformation in VLP vaccines. This conformation directly determines their capacity to induce potent, protective neutralizing antibody responses, positioning D-state VLPs as a promising platform for next-generation poliovirus vaccines with improved safety, stability, and immunogenicity profiles.

### 5.2. Antigen Characterization and Properties

The major neutralizing antigenic epitopes of PV reside on the capsid proteins, particularly within the B-C loop (e.g., Residues 96–104) and D-E loop (e.g., Residues 141–152) of VP1, as well as specific regions of VP2 and VP3. These epitopes are predominantly conformation-dependent [17,50,51]. Studies employing techniques such as cryo-electron microscopy single-particle analysis indicate a high degree of structural homology between VLPs and the wild-type virus in these critical epitopes. For example, structural investigation of a yeast-expressed stabilized PV2 VLP (sVLP) demonstrated successful preservation of the native conformation of neutralizing epitopes. Affinity measurements revealed a dissociation constant (Kd) as low as 10^−9^ M for binding to neutralizing antibodies, and a resolution of 3.5 Å further confirmed the close structural agreement in surface protein architecture between the sVLPs and native virions. These findings provide a robust structural and functional basis for the ability of such VLPs to elicit high-level neutralizing antibody responses [33].

Currently, VLPs for three PV serotypes are constructed separately due to serotype-specific antigenicity, linked to subtle surface epitope variations [16]. ELISA data demonstrate strong homologous antibody binding: PV1 VLPs bind 92% to PV1-specific antibodies, 15% to PV2-specific antibodies, and 12% to PV3-specific antibodies; PV2 binds 89% to PV2-specific antibodies, 18% to PV1-specific antibodies, and 14% to PV3-specific antibodies; and PV3 binds 90% to PV3-specific antibodies, 13% to PV1-specific antibodies, and 16% to PV2-specific antibodies [16]. Chimeric VLPs, made via genetic engineering (e.g., swapping antigenic loops), aid epitope study and multivalent vaccine development [17]. This highlights a key challenge for broadly protective PV vaccines. Additionally, antigen thermal stability tests show that engineered VLP mutants maintain better antigen integrity at high temperatures than traditional inactivated vaccines. For instance, PV1 VLPs expressed in *Pichia pastoris* remain stable above 37 °C [27], and the stabilized PV2 mutant sVLP exhibits significantly enhanced thermal stability compared with its wild-type scaffold [33]. This improved stability facilitates storage and transport in regions with limited cold-chain infrastructure, representing a key advantage for next-generation vaccine candidates.

### 5.3. Immune Response and Immunogenicity

#### 5.3.1. Potent Neutralizing Antibody Responses

Poliovirus VLPs induce strong humoral immunity in animal models. Studies in transgenic mice expressing the human poliovirus receptor show that immunization with a thermally stabilized PV2 VLP produced in *Pichia pastoris* elicits serotype-specific neutralizing antibodies at levels comparable with or higher than the current IPV [33]. For instance, serum neutralizing antibody titers in the sVLP group reached 1:5120, compared with 1:2560 in the IPV group; while this reflects a two-fold difference in serial dilution, this disparity was statistically significant (*p* < 0.01). This high-titer neutralizing antibody response is a key correlate of protective immunity. The efficiency of VLPs in activating B cells stems from their particulate structure and repetitive surface antigen arrays, which efficiently crosslink B cell receptors. This enables robust B cell activation and germinal center responses without the need for additional adjuvants [17]. After immunization, germinal center B cell numbers increased 3.5-fold compared with controls (*p* < 0.001). The resulting antibodies primarily target key conformation-dependent neutralizing epitopes on the viral capsid [17,50,51].

#### 5.3.2. Activation of Cellular Immune Responses

In addition to strong humoral immunity, VLPs show significant potential in activating cellular immune responses. Studies indicate that VLPs are efficiently taken up and processed by antigen-presenting cells (APCs). For example, dendritic cells recognize VLPs via pattern recognition receptors, internalize them with uptake rates exceeding 80%, and process them into antigenic peptides. These peptides are then presented to T cells via MHC Class I and II pathways, activating both CD8^+^ and CD4^+^ T cell responses [52]. Animal studies demonstrated that poliovirus VLPs can induce robust virus-specific Th1 helper T cells and [53]. Compared with traditional IPVs, OPVs elicit stronger CD4^+^ T cell responses and broaden CD4^+^ and CD8^+^ T cell recognition of nonstructural proteins, increasing IFN-γ-secreting cells four-fold and significantly enhancing CTL activity [53]. Such cellular immunity—particularly recognition of nonstructural proteins—is crucial for clearing intracellular virus and providing long-term protection, an area where IPV is relatively weak. VLPs probably exhibit a similar trend to OPVs in promoting cellular immunity but with higher safety. Further research reveals that VLPs can act as “self-adjuvants” by activating pattern recognition receptors such as TLR2 and TLR4, which promote APC maturation and secretion of inflammatory cytokines (e.g., IL-12, TNF-α), thereby enhancing T cell priming [52]. This intrinsic adjuvant effect stems from the VLPs’ nanoparticulate structure and highly repetitive antigen epitopes, which effectively stimulate the innate immune system and create a favorable microenvironment for adaptive immunity. Thus, poliovirus VLPs are theoretically capable of not only eliciting potent neutralizing antibodies but also mobilizing cellular immunity, offering more comprehensive and durable protection—an important advantage against potential viral escape or latent infection.

#### 5.3.3. Exploring Mucosal Immunity Potential

Since PV is primarily transmitted via the fecal–oral route, effective mucosal immunity—particularly in the gut—is critical for blocking viral transmission [26]. Mucosal vaccination can elicit a strong protective immune response at the initial site of pathogen entry, while its needle-free administration and reduced cold-chain dependence offer additional practical and economic benefits [54]. Supporting this approach, a study developed transplastomic lettuce lines for oral immunization, producing VP1-based VLPs approximately 22.3 nm in size. Mice primed with IPVs and given three oral boosters of lyophilized lettuce material (20 mg) adjuvanted with squalene, saponin, or both plus antimicrobial peptides showed specific IgG1 and IgA antibodies as well as neutralizing activity [26]. Compared with the OPV, VLPs are non-replicating and thus safer, eliminating the risk of VDPV. However, the key research challenge remains how to achieve mucosal immune protection comparable with that of live-attenuated vaccines without viral replication [54]. Developing effective mucosal immunization strategies is therefore of major strategic importance for the global eradication of poliomyelitis.

### 5.4. Animal Model Immunization Experiments

Preclinical studies in rodent models (e.g., Wistar rats) demonstrate that stabilized poliovirus-like particles (VLPs), produced in systems like yeast and mammalian cells to preserve native antigenic conformation, can induce neutralizing antibody titers comparable with or exceeding those elicited by traditional IPVs [8,13,16]. The immunogenicity of these VLP vaccines is significantly enhanced by adjuvants (e.g., alum adjuvants), which accelerate and amplify the antibody response. These collective findings validate the potential of VLP-based candidates as safe, effective, and scalable alternatives for future immunization strategies.

## 6. Cryo-Electron Microscopy (Cryo-EM) Structural Analysis

High-resolution cryo-EM has become indispensable for resolving the three-dimensional (3D) architecture of PV VLPs, demonstrating their striking structural mimicry of native infectious virions [33]. Cryo-EM studies show that stabilized VLPs, recombinantly produced stabilized VLPs (e.g., in *Pichia pastoris*), closely mimic the native D-antigen conformation, which is essential for eliciting potent neutralizing antibodies [8]. For instance, cryo-EM reconstructions of thermally stabilized PV3 VLPs (PV-3 SC8) exhibit a capsid architecture nearly identical to infectious PV3, underscoring their potential as next-generation vaccines with enhanced thermostability and immunogenicity [8]. Similarly, cryo-EM studies revealed that PV2 sVLP maintains a native-like compact conformation, whereas the wild-type VLPs adopt a non-native expanded state correlating with reduced immunogenicity [33]. These insights highlight that structural integrity is a critical determinant of vaccine efficacy and that cryo-EM is a vital tool for verifying the preservation of the authentic viral architecture in VLP candidates.

High-resolution cryo-EM, beyond confirming structural mimicry, underpins how specific stabilizing mutations boost VLPs’ stability and antigenic epitope preservation. Key among its insights is the identification of a conserved GSH-binding pocket at the PV capsid interprotomer interface via cryo-EM, where GSH binding enhances particle stability by reinforcing the interprotomer interactions critical for capsid integrity—findings that inform the rational design of antiviral compounds or vaccine stabilizers mimicking GSH to lock the capsid in a stable conformation. Supporting this, cryo-EM analysis of PV2 VLPs bound to benzene sulfonamide compounds showed that these molecules similarly occupy the interprotomer pocket, over-stabilizing the capsid and abrogating infection-related conformational changes [55]. Cryo-EM comparisons of PV2-stabilized mutants (e.g., SC5a, SC6b) have also revealed subtle conformational differences correlating with enhanced immunogenicity and yields in yeast fermentation, enabling VLP design optimization by pinpointing mutations that preserve neutralizing epitope presentation while improving stability during manufacturing and storage [47].

Collectively, cryo-EM has not only confirmed the high 3D similarity between stabilized VLPs and native PV but has also elucidated the molecular basis by which specific interactions and mutations drive VLPs’ stability and antigenic fidelity. This mechanistic understanding underpins the development of next-generation poliovirus vaccines that are safer, more stable, and potentially more immunogenic than current inactivated vaccines, advancing global polio eradication efforts [8,33,47,55].

## 7. Application Prospects and Challenges of Poliovirus VLP Vaccines

### 7.1. Safety and Production Advantages

PV VLP vaccines present significant safety and production advantages over traditional vaccines Primarily, their lack of viral genomic material eliminates the risks of pathogenic reversion, environmental transmission, and vaccine-derived PV emergence of VDPVs [23,27]. This inherent biosafety permits scalable production in heterologous expression systems (e.g., yeast or mammalian cells) without the need to handle live, infectious viruses [8,27,47].

### 7.2. Vaccine Immunization Strategies and Global Eradication Programs

VLP vaccines offer a critical safety profile for global eradication programs, as their non-infectious, replication-deficient nature eliminates the risk of vaccine-associated paralytic poliomyelitis and VDPV outbreaks—a persistent concern with the OPV. This makes VLPs ideal for sustaining population immunity after OPVs’ cessation [56]. Moreover, they can induce robust systemic humoral responses and, with an appropriate formulation, hold the potential to enhance mucosal immunity, thereby addressing a key limitation of IPVs [57]. Successful eradication requires sustained high coverage, vigilant surveillance, and rapid outbreak response capacity. VLP vaccines, with their inherent safety, potential for thermostability, and scalable production free from live virus risks, are a promising platform for post-eradication immunization. Integrating them with other novel platforms (e.g., VLPs, nOPVs, IPVs, mRNA vaccines) into comprehensive immunization schedules is essential for achieving and maintaining a polio-free world [2,13,33,56,57].

### 7.3. Immune Persistence and Adjuvant Optimization

Further investigation into poliovirus VLP vaccines’ long-term immune persistence and adjuvant formulation optimization is critical for enhancing efficacy and durability, with current evidence highlighting adjuvant choice (e.g., aluminum-based, W/O/W emulsions, nanoparticles) as a key factor in boosting immunogenicity, though longitudinal data on immunity duration remain limited [33,58,59].

Future research should focus on a systematic evaluation of adjuvant candidates, innovative delivery platforms, and the mechanistic interplay of VLPs’ structure, adjuvants, and host immunity to develop optimized next-generation vaccines supporting sustained global polio eradication [17,60,61].

### 7.4. Multivalent and Combined Vaccine Development

The development of multivalent VLP vaccines targeting multiple PV serotypes and other enteroviruses is imperative, given the limited cross-protection of monovalent vaccines against diverse enterovirus genotypes, with VLPs serving as a safe and immunogenic platform for multi-antigen display (e.g., VLPs as multivalent vaccine candidates against Chikungunya, Japanese encephalitis, yellow fever and Zika virus) [62]. These vaccines also can integrate antigens from other pathogens, offering substantial public health benefits including the reduced incidence of related diseases, streamlined immunization programs, and improved biosafety, while ongoing optimization is crucial to fully realize their potential in enhancing global public health outcomes [13,16,47].

### 7.5. Clinical Transformation Progress

The world’s first PV VLP vaccine, developed by CanSino Biologics, started Phase I clinical trials in Australia (January 2024, first subject enrolled) and obtained Indonesian regulatory approval for Phase I/II trials (October 2024), which launched in December 2024 to assess safety and immunogenicity in infants. Invited to the WHO Annual Consultation Meeting on Global Polio Vaccines on World Polio Day 2025, CanSino presented the vaccine’s R&D progress. With proprietary, live virus-free VLP assembly technology, this vaccine is listed by WHO as a preferred candidate for global polio eradication and has secured two rounds of funding from the Bill & Melinda Gates Foundation to expedite its global development [63]. The established benchmarks have been met, encompassing the completion of rodent immunogenicity studies with neutralizing antibody titers that satisfy predefined thresholds, the execution of fundamental safety assessments, and the optimization of production processes with yields and purity conforming to regulatory specifications. However, several critical aspects remain to be addressed, including long-term toxicity data in non-human primates, the combined immunization efficacy of multivalent PV1/PV2/PV3 vaccines, safety evaluations in special populations (e.g., infants with specific constitutions), and the validation of quality control systems for large-scale manufacturing.

Furthermore, foundational research on PV VLP vaccines leveraging diverse expression platforms is actively underway both domestically and internationally (Table 2) [13,16,23,25,27,33,47,64,65]. The collective evidence positions VLP-based strategies as one of the most promising next-generation approaches to advance polio’s elimination and sustain a polio-free status globally.

## 8. Conclusions

In conclusion, PV VLP vaccines represent a promising next-generation immunization platform, integrating high safety, robust immunogenicity, and manufacturing feasibility. Driven by interdisciplinary advances in molecular biology, structural virology, and vaccine technology, these VLPs are rationally designed to mimic native virion structures without replication or reversion risks, enabling precise antigen presentation and potent, durable immune responses. Such innovations address the inherent limitations of traditional poliovirus vaccines, underscoring their pivotal potential in sustaining global immunity during and after polio’s eradication.

Nevertheless, critical challenges remain, including optimizing scalable and cost-effective manufacturing processes to meet global demand, enhancing the durability of induced immune responses to match or surpass existing vaccines, and refining multivalent formulations to ensure comprehensive serotype coverage while preserving stability and immunogenicity.

To fully realize the translational potential of PV VLPs vaccines, future research should prioritize enhancing vaccines’ stability under diverse storage and distribution conditions, extending the duration of protective immunity, and optimizing multivalent designs. Integrating findings from immunology, bioprocess engineering, and clinical evaluations through interdisciplinary collaboration will accelerate bench-to-bedside translation, facilitating equitable global access—particularly in resource-limited settings. Ultimately, the continuous evolution of poliovirus VLP vaccines aligns with the global health objective of a polio-free world, offering a safe, effective, and adaptable strategy to consolidate eradication gains and prevent resurgence.

## Figures and Tables

**Table 1:** Mutations that suppress the effect of the capsid-destabilizing mutation in PV.

Serotypes	Reference Strains	Mutations	Location	Strains, Where Identified	References
Type 1	VS51	VP4: F4046L	Internally located in the virus capsid	/	[24]
		VP1: T1026A	Internal surface of the virus capsid; close to the N-terminus of VP1	/	[24]
		VP3: T3175A	Surface G-H loop of the VP3 β-barrel; a capsid surface residue located on the surface G-H loop of the VP3 β-barrel;	/	[24]
	Mahoney-SC7	R4018G *	Internal network near a three-fold axis	/	[39]
		T2025A *	Pentamer interface	/	[39]
		D2057E *	Pentamer interface	/	[39]
		L3119M *	VP2/VP3 interface	/	[39]
		Q3178L *	Protomer interface	/	[39]
		V1196L *	Pocket	/	[39]
		H1248P *	Protomer interface	/	[39]
Type 2	MEF-SC5a	L3085F	Beta sheet at the pentamer interface	/	[39]
		Q3178L	Pentamer interface	/	[39]
		T1041I	Pentamer interface	/	[39]
		F1134L	Pocket	/	[39]
		Y1159F	Pocket	/	[39]
Type 3	Sabin 3	VP2: L18I	Beta sheet at the pentamer interface	Isolates from vaccinees and in vitro passage at elevated temperatures	[39]
		VP2: L215M	Protomer interface	Isolates from vaccinees	[39]
		VP2: D241E	VP2/VP3 interface, buried	In vitro passage at elevated temperatures	[39]
		VP3: H19Y	VP2/VP3 interface, buried	In vitro passage at elevated temperatures	[39]
		VP3: L85F	Beta sheet at the pentamer interface	In vitro passage at elevated temperatures	[39]
		VP3: F91S	Protomer interface	Isolates from vaccinees and in vitro passage at elevated temperature	[39]
		VP1: A54V	Internal network at a three-fold axis	Isolates from vaccinees and in vitro passage at elevated temperatures	[39]
		VP1: F132L	Capsid pocket	In vitro passage at elevated temperatures	[39]
	Saukett-SC8	T4067A *	Internal network near a three-fold axis	/	[39]
		L2018I *	Beta sheet at the pentamer interface	/	[39]
		L2215M *	Protomer interface	/	[39]
		D2241E *	VP2/VP3 interface, buried	/	[39]
		H3019Y *	VP2/VP3 interface, buried	/	[39]
		L3085F *	Beta sheet at the pentamer interface	/	[39]
		T1105M *	North wall of the canyon	/	[39]
		F1132L *	Pocket	/	[39]

“*” denotes residues that still require further research and validation; “/” indicates that the content is not applicable or not stated in the cited literature.

**Table 2:** List of studies on poliovirus VLP vaccines.

No.	Serotypes	Composition	Expression System	Immunization Route	Key Animal Study Results/Conclusions	Year of Publication	References
1	PV1	P1-3CD	HeLa	/	(1) P1 was successfully treated correctly, and three capsid proteins (VP0, VP3, and VP1) were produced. (2) Its size and shape are consistent with the expectation of the empty capsid of poliovirus.	1991	[64]
2	/	P1-3CD	Yeast: Saccharomyces cerevisiae	/	(1) N-antigenicity empty capsid can be separated from the yeast cell extract. (2) The purified empty capsid has the same immunogenicity as poliovirus particles.	1997	[65]
3	PV3 (PV3 SktSC8 mutant)	P1-3CD	Plant: Nicotiana benthamiana	I.P.	(1) Mice expressing the human PV receptor gene are protected against wild-type PV infection following immunization with plant-derived PV sVLPs. (2) Single-particle reconstruction reveals a structure almost indistinguishable from wild-type PV3.	2017	[25]
4	PV1 (Mahoney strain), PV2 (MEF-1 strain), PV 3 (Saukett strain)	Strategy I: P1-3CD; Strategy II: VP1-VP3-2A-VP0	Insect cell line Sf9 (recombinant baculovirus vector)	I.P.	(1) Strategy II was adopted for the subsequent production of PV-VLPs, as it yielded higher levels of structural proteins of PV than Strategy I. (2) The number of D-antigen units displayed by PV-sVLPs was more than 3.9-fold higher than that displayed by the same quantity of PV-VLPs. (3) Introduction of the designed mutations significantly enhanced the thermostability of the VLPs. (4) Compared with an equivalent amount of PV-VLPs, PV-sVLPs exhibited more D-antigens and elicited higher neutralizing antibody titers. (5) Splenocytes isolated from immunized mice secreted high levels of IFN-γ, IL-2, GM-CSF, IL-5, and IL-10 following stimulation with PV-sVLPs.	2019	[66]
5	WPV1 (Mahoney strain)	P1-3CD	Yeast: *Pichia pastoris*	/	(1) The dual promoter expression system represents the most efficient approach for generating picornavirus VLPs. (2) The *Pichia expression* system serves as an efficient platform for the production of D-antigens.	2020	[23]
6	PV1 (Mahoney strain), PV2 (MEF-1 strain), PV3 (Saukett strain) and the 3 CD sequence (PV1 Mahoney strain)	P1-3CD (no self-cleavage of 3CD into 3Cpro and 3Dpol)	Mammalian cells	I.M.	(1) The 3CD sequence contained a mutation at the cleavage site between 3C and 3D: a Ser was inserted after Residue 181 of 3C, which prevented self-cleavage of 3CD into 3Cpro and 3Dpol to form 3CD.	2021	[13]
7	PV1 SC6b Mahoney	P1-3CD	Yeast: *Pichia pastoris*	/	(1) These VLPs (by separately expressing P1 and 3CD) were thermostable above 37 °C, demonstrating their potential as next-generation vaccine candidates for PV. (2) Both VP3-P2A constructs yielded D-antigen-reactive particles with hardly any C-antigen detected, indicating that the C-terminal tag on VP3 does not impair the antigenicity of the assembled particles. (3) The antigenic stability of protease-free PV VLPs at physiologically relevant temperatures indicates their potential to elicit long-term protective immunity against poliovirus.	2023	[27]
8	PV1 Mahoney, PV2 MEF-1 and PV3 Saukett	P1-3CD	BHK-21 cells; mammalian, baculovirus, yeast, plant	I.P. or I.M.	(1) Using four distinct recombinant expression systems, poliovirus VLPs of all three serotypes were successfully produced, and these stabilized VLPs exhibit antigenicity, thermostability, and immunogenicity equivalent or superior to the current IPV in female Wistar rats. (2) The rsVLPs generated from all four expression systems retain stability above 40 °C; the PV1-SC6b rsVLPs produced in yeast, plant, and insect cells exhibit reduced thermal stability compared with the IPV. (3) Except for PV1-SC6b produced in mammalian cells via MVA, rsVLPs were detected in the D-antigen conformation across all serotypes and expression systems. (4) Compared with IPV, all rsVLPs induced neutralizing antibody titers that were equivalent to or higher than those elicited by the reference vaccine.	2025	[16]
9	PV1-SC6b, PV2-SC6b, and PV3-SC8	P1-3CD	Yeast: *Pichia pastoris*	I.P.	(1) Trivalent immunogenicity assays with all three serotypes of bioreactor-derived VLPs in the presence of Alhydrogel adjuvant demonstrated that these VLPs outperform the current IPV vaccine in standard vaccine potency analyses, offering dose-sparing potential. (2) The PV2-stabilized mutant SC5a improves VLP yield in both flask and bioreactor production. (3) The structure of the PV2 SC6bVLPs from yeast bioreactor material is identical in its overall features and conformation to the same VLPs produced in mammalian and insect cells. (4) Trivalent immunogenicity assays with all three serotypes of bioreactor-derived VLPs in the presence of Alhydrogel adjuvant demonstrated that these VLPs outperform the current IPV vaccine in standard vaccine potency analyses, offering dose-sparing potential.	2025	[47]
10	PV2 wt strain MEF-1 (AY238473.1) and the MEF-SC5a mutant	VP0-VP1-VP3 fragments	Yeast: *Pichia pastoris*	I.P.	(1) This confirms the successful co-expression and assembly of VP0, VP3, and VP1, circumventing the potential toxicity of 3CD. (2) The results demonstrated that 1 µg of PV2 sVLPs contained 7.33 units of D-antigen, in contrast to only 2.79 units in PV2 wtVLP. (3) PV2 sVLPs were observed to be superior to wtVLPs in terms of the thermal stability of the D-antigen. (4) Pseudovirus-based neutralization assay.	2024	[33]

Note: I.P.: intraperitoneal injection; I.M.: intramuscular injection.

## Data Availability

Not applicable.

## Abbreviations

The following abbreviations are used in this manuscript.

PV	Poliovirus
OPV	Poliovirus vaccine
IPV	Inactivated poliovirus vaccine
VLP	Virus-like particle
GPEI	Global Polio Eradication Initiative
WPV1	Wild-type poliovirus Type 1
WPV2	Wild-type poliovirus Type 2
WPV3	Wild-type poliovirus Type 3
VDPVs	Vaccine-derived polioviruses
ORF	Open reading frame
ECs	Empty capsids
VAPP	Vaccine-associated paralytic poliomyelitis
cVDPV	Circulating vaccine-derived PV
nOPV2	Novel oral poliovirus Type 2
IRES	Internal ribosome entry site
RhPV	Rhopalosiphum padivirus
GSH	Glutathione
Cryo-E	Cryo-electron microscopy
wt	Wild-type
CTLs	Cytotoxic T lymphocytes
